# The Effectiveness of the Buzzy Device in Reducing Pain in Children Undergoing Venipuncture: A Single-Center Experience

**DOI:** 10.1097/PEC.0000000000003011

**Published:** 2023-07-22

**Authors:** Elisa Simoncini, Giulia Stiaccini, Elena Morelli, Elisa Trentini, Diego G. Peroni, Maria Di Cicco

**Affiliations:** From the ∗ Paediatrics Unit, Pisa University Hospital, Pisa, Italy; † Department of Clinical and Experimental Medicine, University of Pisa, Pisa, Italy.

**Keywords:** adolescence, childhood, pain management, phlebotomy, procedural pain

## Abstract

**Objectives:**

Needle-related procedures are among the most important sources of pain in children in different health care settings. Our study was aimed to evaluate the effectiveness of Buzzy (MMJ Labs, Atlanta, Ga.), a palm-sized bee/ladybug-shaped device combining vibration and cold, as a nonpharmacological strategy to manage needle-related pain in children.

**Methods:**

In this single-center, randomized (1:1) controlled open-label study, we enrolled patients aged from 1 month to 18 years who had to undergo a planned outpatient blood sampling in Pisa University Hospital's Department of Pediatrics and randomly allocated them to either the BUZZY group (intervention group) or NO BUZZY group (control group). Pain was estimated using proper pain scales according to age.

**Results:**

Between May 2021 and January 2022, 234 children aged 8.8 ± 5.1 years (50.8% girls) were enrolled and 117 were treated with the Buzzy device. In the study population, pain inversely correlated with age (r = −0.52, *P* < 0.001); the intervention group showed significantly lower pain (2.5 ± 2.4 vs 4.7 ± 2.8, *P* < 0.001) and no difference was found between boys and girls. Significant reduction in pain scores was confirmed when stratifying children by age (29 days to <3 years, *P* = 0.002; ≥3 to ≤8 years, *P* < 0.001; >8 years, *P* < 0.001).

**Conclusions:**

The Buzzy device effectively reduces pain caused by percutaneous antecubital venipuncture in children in different age groups and represents a cheap and easy-to-use strategy to manage routine needle-related procedures.

Needle-related procedures, such as venipuncture, injections, and intravenous cannulation, are considered as the most important sources of fear, pain, and distress in children in different health care settings.^1–3^ Notably, procedural pain in childhood could result in physiological, psychological, and emotional consequences such as increased pain sensitivity and inappropriate pain responses, worsened by subsequent procedures in a vicious cycle fashion.^4^ Children may also show anticipatory anxiety, which can generate physiological symptoms during needle-related procedures, such as vasovagal reactions, hypoxemia, tachycardia, and changes in hormone levels.^5^ Lastly, children may develop needle phobia, which may lead to avoidance of vaccination, resistance to blood examination, and poor compliance, thus hampering adequate health care.^6,7^ Such attitudes may persist even in adolescence and in adulthood.^8^ Therefore, the use and optimization of pain management interventions during these procedures is of pivotal importance in childhood.^9^ Several pharmacologic and nonpharmacologic strategies are currently available: among others, skin application of topical anesthetics 30 to 60 minutes before blood collection is commonly used, but such a strategy may be expensive and not optimal in settings in which time is short and the workload heavy. Nevertheless, techniques such as J-tip and vapocoolant sprays provide faster topical analgesic effect, but the former may not be available or authorized outside the United States. Nonpharmacological strategies of pain control, such as distraction techniques, are cheap, safe, and easy to use, but also time-consuming and require trained health care professionals.^10–12^ Among these techniques, those combining the involvement of multiple senses are particularly effective. As a matter of fact, it is well known that vibration, cold, and touch can interfere with pain nervous transmission. The Buzzy device (MMJ Labs, Atlanta, Ga.) is an 8 × 5 × 2.5 cm plastic reusable bee- or ladybug-shaped, palm-sized device that was created by a pediatrician, Dr. Amy Baxter, and introduced to the market in 2009 as a new strategy to reduce needle-related pain in children.^13^ The device has 2 components: (i) the body of the bee/ladybug with a vibration motor powered by 2 alkaline AAA batteries providing continuous or intermittent vibration when activated and (ii) a detachable wing-shaped cooling pad containing nontoxic gel, which can remain frozen for approximately 10 minutes at room temperature and should be placed on the patient's skin proximal to the procedure site (Fig. 1). The Buzzy device interferes with pain by the following proposed mechanisms: a) distraction, b) gate control theory, c) descending noxious inhibitory controls (DNIC), d) local analgesia due to numbness caused by vibration and release of endogenous opioids caused by the activation of cold-related touch receptors. According to the gate control theory, pain is conducted from the peripheral nervous system to the central nervous system via modulation through a gating system in the dorsal horn of the spinal cord. Vibration on the skin blocks afferent pain-receptive nerves (C and A-delta fibers) by stimulating A-beta nerve fibers (fast nonnociceptive motion nerves), which activate an inhibitory interneuron, resulting in a reduction of the pain signal transmitted to the spinal cord, eventually closing the pain gate.^14^ On the other hand, the cold component excites C fibers, sending noxious thermal information to the brain and further blocks the A-delta pain signal when applied before the pain stimulus^13^ (Suppl. Fig. 1, http://links.lww.com/PEC/B129). According to the descending noxious inhibitory controls theory, cold stimulates C fibers and activates a descending supraspinal modulation, which raises the body's overall pain threshold, thus producing a generalized hypoalgesia at the insertion site.^14,15^ Several random randomized clinical trials have been conducted to evaluate the Buzzy device during different needle-related procedures in children, mostly confirming its efficacy in pain management, as shown in a systematic review focused on the device published in 2019, which included 9 studies involving 1138 participants aged between 3 and 18 years.^16^ The aim of this single-center study carried out in the pediatric blood-drawing center of an Italian university hospital was to further assess the effectiveness of the Buzzy device in controlling venipuncture-related pain in children and adolescents undergoing routine outpatient blood collection, to add more data on the subject, considering the paucity of trials already conducted and the limited age range usually considered in these previous studies.

**FIGURE 1:**
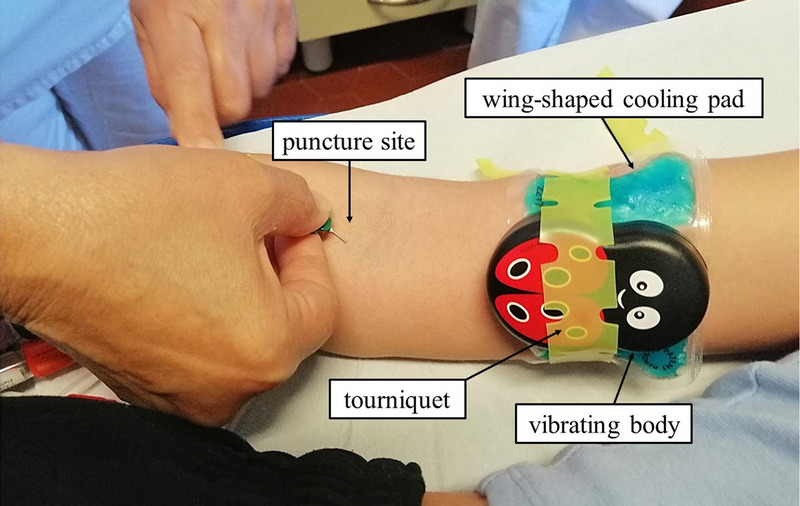
The figure shows how the Buzzy device was placed and used in the study. Details are provided in the text. Note that we chose the ladybug model to avoid potential fear of bees.

## METHODS

### Study Design and Participants

This study was designed as a prospective randomized (1:1) open-label study. Between May 2021 and January 2022, we enrolled patients aged between 29 days and 18 years who had to undergo a planned routine outpatient blood sampling at the Pediatrics Unit of Pisa University Hospital, Pisa, Italy, dividing them into 3 age groups (29 days to <3 years, ≥3 to ≤8 years, >8 years). While the patients were waiting for venipuncture, the study's aims and methods as well as the Buzzy device functioning were explained to the parents and to older children. If they agreed to participate, written consent was obtained from at least 1 parent and from children older than 12 years, together with general information. Children undergoing intravenous cannulation or invasive procedures different from venipuncture were excluded, together with those refusing to wear the device or with significant needle phobia. We also excluded those who knew or had already used the Buzzy device, as well as those with bleeding disorders; presence of infection, rash, abrasion, or generally damaged skin in the area where the device would be placed; neurodevelopmental or behavioral disorders; critically ill or unstable patients; use of topical, enteral, or parenteral analgesics within 24 hours of presentation; nerve damage or peripheral neuropathy in the area of venipuncture, and cold hypersensitivity diseases such as Raynaud disease and Prader-Willi syndrome. If the child was eligible and the informed consents were signed, for each age group, patients were randomly assigned to either the BUZZY group (venipuncture with Buzzy) or the NO BUZZY group (venipuncture without Buzzy). Both enrollment and randomization were stratified by age; randomization was based on every other patient, and every child was randomized (1:1) in each age group. Venipuncture was performed by means of the Vacutainer (BD Vacutainer, Franklin Lakes, NJ) system, using a 21G butterfly needle. All venipuncture procedures were performed by the same nurse with more than 5 years' experience in pediatric assistance and venipuncture, who had received information and practical training in the use of the Buzzy device. Each child could participate in the study only once.

### The Buzzy Device

For the purposes of our study, we chose to use the ladybug-shaped Buzzy (Buzzy Healthcare mini ladybuzz, code BKHM3). The wings were kept in the freezer before the procedure and were taken out a few minutes before application on the skin, so that the patient did not feel the discomfort of excessive cold. It should be noted that the use of the wings was mandatory in our study to evaluate the effect of combined cold and vibration. Once the child and the nurse were ready, the frozen wings were inserted into the back of the ladybug's body through fixed elastic bands. Then, the Buzzy device was applied on the patient's skin 5 cm above the planned puncture site on the forearm (Fig. 1) and secured with a dedicated tourniquet. Continuous vibration was activated by a manual switch located at the top of the device approximately 30 seconds before venipuncture; after blood sampling, the Buzzy device was left in place for approximately 15 to 30 seconds and then turned off and removed. After the procedure, the cooling pad was thoroughly wiped with 70% alcohol and then put back in the freezer to be used for the following patient. The total intervention time was approximately 2 minutes. The parents in both groups accompanied their child throughout the procedure and were actively involved in supporting and consoling their child.

### Pain Scoring Scales

Immediately after venipuncture, children older than 3 years were asked to estimate the level of experienced pain using a proper scale, selected according to the age of the patient, whereas in younger children, it was the same nurse trained in the use of behavioral scale who rated the pain. The nurse was not blinded to the purpose of the study. Among the more than 50 scales currently used to measure children's pain,^17^ we chose those that are most used and considered as reliable, valid, and with internal consistency in different age groups (Suppl Table 1, http://links.lww.com/PEC/B130): The Face, Legs, Activity, Cry, and Consolability scale^18^ was used in patients younger than 3 years, whereas the Wong-Baker FACES scale was used in children aged from 3 to 8 years, and the Numeric Rating scale was used in children from 8 to 18 years.^19^ Children and parents in the BUZZY group could also choose whether to leave an anonymous written open-ended comment on the procedure.

### Data Collection and Study Outcomes

Demographical data, site of the venipuncture and number of attempts, pain scores, complications (such as vasovagal reaction), and parents' and children's comments were recorded using a proforma. Data relevant to the study were recorded and analyzed anonymously. The primary outcome was pain evaluation in children undergoing routine blood testing, with or without the Buzzy device. This study was approved by our hospital's ethics committee and by our department's review board. This study was carried out in accordance with the Declaration of Helsinki, International Conference on Harmonisation Good Clinical Practice guidelines, and under the terms of all relevant local legislation.

### Statistical Analysis

The data are presented as number, percentage, and mean ± SD or median and interquartile range when appropriate. Differences of categorical variables were analyzed using χ^2^ test or, where the sample size was small, using Fisher exact test. Differences between means were determined using unpaired Student *t* test, and those between medians using Mann-Whitney test, when appropriate. The association between children's age and pain scores was analyzed with the Pearson correlation coefficient. A *P* value of <0.05 was considered significant. The analysis was carried out using the SPSS (Statistic Package for Social Science) software version 21.0 (IBM Corp., Armonk, NY).

## RESULTS

**FIGURE 2:**
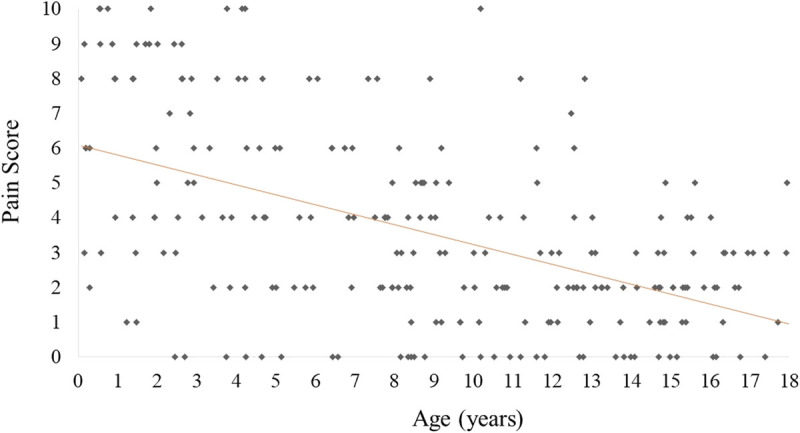
Correlation between age of the subjects and pain scores (r = −0.518, *P* < 0.001).

**FIGURE 3:**
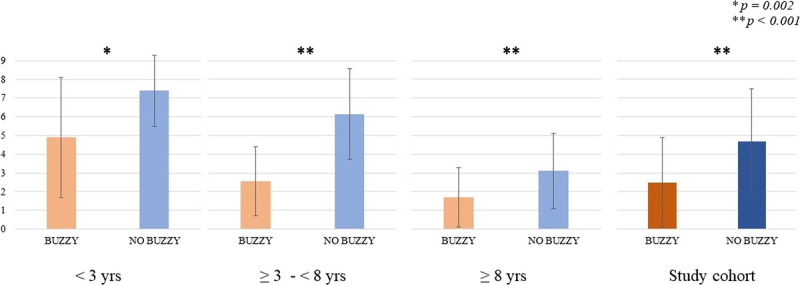
Pain scores (mean ± SD) in the study cohort and in the 3 age groups; we found significantly lower scores in the BUZZY groups. Note that pain scores are higher in younger children, in agreement with the correlation analysis.

In the study period, 234 children aged 8.8 ± 5.1 years (range: 1 month–17.9 years), 119 of whom were girls (50.8%) were enrolled in the study; all patients underwent percutaneous antecubital venipuncture. The 3 age groups (0 and <3 years, between ≥3 and <8 years, ≥8 years) included 46 (19.6%), 54 (23.1%), and 134 (57.3%) patients, respectively. Every child was randomized (1:1) so that 117 patients were included in the BUZZY group and the other 117 in the NO BUZZY group; the children's mean ages were 8.9 ± 5.1 years and 8.7 ± 5.1 years in the intervention and control groups, respectively (*P* = 0.703) (Table 1). Venipuncture was successful at the first attempt in 228 patients (97.4%). Among the remaining 6 cases, 5 children required 2 attempts and only 1 child, aged 8.7 years, underwent 3 attempts; only 1 child required 2 attempts in the BUZZY group. During or immediately after venipuncture, 3 children had a vasovagal episode, reporting they were feeling unwell and showing transient pallor, profuse sweating, and hypotension. These patients were all aged between 9 and 18 years, 2 were from the BUZZY group, and 1 underwent 2 attempts. No technical difficulty was recorded during the use of the Buzzy device. In the whole study population, we found an inverse correlation between mean pain scores and age (r = −0.518, *P* < 0.001) (Fig. 2). The average pain for the venipuncture was 2.5 ± 2.4 (range, 0–10) in the BUZZY group and at 4.7 ± 2.8 points (range, 0–10) in the NO BUZZY group (*P* < 0.001). No difference was found between boys and girls (Table 1). Significant differences were confirmed when stratifying children into 3 age groups (Fig. 3). Among those in the BUZZY group, 31 children aged 12.8 ± 3.5 years and the parents of 14 children aged 4.1 ± 2.0 years left anonymous written comments on their experience with the Buzzy device; 13 parents referred that they felt their children had experienced less pain and fear than in previous blood samplings. One parent referred that it was the first time that her child had had to undergo a venipuncture and felt that the Buzzy device helped. Among the children, most of them wanted to share they had felt less pain than in previous venipunctures; 5 of them added that they attributed the efficacy of the Buzzy to the vibration, which was felt as a massage, more than to the cooling pad.

**TABLE 1:** Study Population

	BUZZY Group	No BUZZY Group	*P*
Overall, n (%)	117 (50)	117 (50)	
F (n)	56 (47.9)	63 (53.8)	0.360
Age (M ± SD)	8.9 ± 5.1	8.7 ± 5.1	0.703
Pain score (M ± SD)	2.5 ± 2.4	4.7 ± 2.8	**<0.001**
Age groups			
<3 y, n (%)	23 (19.7)	23 (19.7)	
F (n)	12 (52.2)	10 (43.5)	0.554
Age (M ± SD)	1.8 ± 0.7	1.4 ± 1.0	0.097
Pain score (M ± SD)	4.9 ± 3.2	7.4 ± 1.9	**0.002**
≥3 to <8 y, n (%)	27 (23.1)	27 (23.1)	
F (n)	12 (44.4)	16 (59.3)	0.273
Age (M ± SD)	5.4 ± 1.5	5.7 ± 1.5	0.551
Pain score (M ± SD)	2.6 ± 1.8	6.1 ± 2.4	**<0.001**
≥9 y, n (%)	67 (57.3)	67 (57.3)	
F (n)	32 (47.8)	37 (55.2)	0.387
Age (M ± SD)	12.7 ± 2.7	12.4 ± 3.0	0.563
Pain score (M ± SD)	1.7 ± 1.6	3.1 ± 2.0	**<0.001**
Sex			
Males, n (%)	61 (26.0)	54 (23.1)	
Age (M ± SD)	8.6 ± 5.0	8.3 ± 5.2	0.745
Pain score (median, IQR)	2 (0–4)	5 (2–8)	**<0.001**
Females, n (%)	56 (23.9)	63 (27.0)	
Age (M ± SD)	9.1 ± 5.2	9.0 ± 5.1	0.478
Pain score (M ± SD)	2.7 ± 2.3	4.3 ± 2.6	**<0.001**

Bold values denote statistical significance at the *P* < 0.05 level.

IQR indicates interquartile range; M, mean.

## DISCUSSION

In this single-center study, we found that the Buzzy device represents an effective strategy to manage pain during routine venipuncture in children from different age groups. Our study adds evidence to the already available evidence on the subject, including several randomized clinical trials carried out in children mostly in outpatient settings.^16,20^ The device was found effective when compared with standard care also in the setting of pediatric emergency departments.^13^ The available evidence suggests that the Buzzy device is effective also in children undergoing peripheral intravenous cannulation,^21,22^ intramuscular injections,^23,24^ routine vaccine injections,^25^ insulin injections,^26^ and even dentistry procedures.^27^ The device was effectively used also in children with cognitive impairment.^28^ Although the Buzzy seems more effective than other nonpharmacological techniques,^29–32^ the same may not be true for pharmacological strategies, as shown by studies providing conflicting results.^33–35^ Nevertheless, even if recent data suggest that anesthetic patches are more effective than the Buzzy device,^36^ such a strategy may be more expensive and requires almost 1 hour to obtain an optimal analgesic effect, thus potentially limiting its usefulness in the clinical practice in settings where time is short and the workload heavy, such as in hospitals' blood-drawing centers. However, it should be noted that most of these studies were focused on specific age groups (school-aged children, adolescents), whereas our study included all pediatric age groups. Notably, the systematic review published in 2019 included studies involving only patients aged older than 3 years.

Whether the Buzzy device is effective due to vibration, its cooling effect, or both, given the evidence on the efficacy of both of them, is still a matter of debate.^37,38^ Moreover, part of its effect may be due to its distractive feature, especially in younger children. Even if evaluating the different analgesic components of the Buzzy device was beyond the goals of our study. It should be noted that among the children in the BUZZY group who left a comment on the device, 5 attributed its efficacy to the vibration.

Even if our study was not designed to evaluate the role of sex and age in pain perception, it should be noted that we found no difference in scores between boys and girls as well as an inverse correlation between pain scores and age. These data agree with evidence from the literature showing that the threshold of pain remains almost the same between boys and girls at least up to the age of 14,^39^ with a reported increase of pain threshold with age. As a consequence, the Buzzy device may be particularly useful in younger children, but it should be remembered that it has been reported that more than 25% of subjects older than 12 years report significant distress associated with venipuncture.^40^ Nevertheless, one of the strengths of our study is that, unlike most of other studies carried out so far, we chose to include children of all ages undergoing routine venipuncture, including very young patients (namely, those aged younger than 3 years), who are those experiencing the worst pain and anxiety during invasive procedures. Considering that the Buzzy device is cheap and easy to use and could be introduced in blood-drawing centers as a routine procedure, we sought to evaluate a real-life condition, with daily routine procedures carried out on children of all ages irrespective of their previous experiences with venipuncture, which could have influenced their pain responses. For the same reason, we chose not to select the patients on the basis of race, although we are aware of the fact that cultural and racial factors can influence the experience of pain. Notably, all the parents involved agreed to participate and were glad to try the Buzzy device, hoping that it could make the procedure less painful for their children. Moreover, to our knowledge, our study is the first to evaluate ladybug-shaped Buzzy device rather than the bee-shaped one. We chose this particular model considering that bees may be frightening for some children.

Regarding the venipuncture procedure, no technical problem was reported by the study nurse, and among the 6 cases in which more attempts were needed, only 1 was in the BUZZY group, and the nurse did not attribute that issue to the Buzzy device.

Our study has several limitations. First of all, it was impossible to lead a blind trial due to the features of the Buzzy device; therefore its efficacy may be related, at least in part, to the placebo effect. Moreover, we had to apply different pain scales to our patients based on the child's age and cognitive level, which means that their scores are not directly comparable. Even if we chose the most used scales worldwide, the Face, Legs, Activity, Cry, and Consolability scale is based on behavioral reactions evaluated by an experienced health professional, which could potentially poorly correlate with the actual perceived pain, whereas the Wong-Baker FACES scale and Numeric Rating scale are based on self-reporting pain intensity. It should also be noted that the reliability of pain scales can be influenced by children's emotional factors and that some children may confuse pain with fear. Even though we found a significant reduction in pain scores both when evaluating the whole study cohort and when stratifying children by age group, we must admit that the groups aged 0 to 3 and 3 to 8 years do not include a substantial number of patients, but this reflects the typical pediatric department activity, where younger children do not usually need routine blood testing very frequently. Moreover, we did not evaluate whether the use of Buzzy might represent a preanalytic issue, which may hamper the interpretation of the laboratory results, considering that direct skin cooling causes vasoconstriction and hemoconcentration, potentially increasing the concentration of some blood analytes at the puncture site. Vibration promoted by Buzzy could also influence blood analytes due to its potential effect on muscles. As a matter of fact, in a recent article by Lima et al,^41^ blood was collected from 100 adult volunteers with or without the Buzzy device, showing significant differences in total proteins, albumin, and transferrin levels; therefore, the authors suggest using the device with caution when these analytes are requested. More studies are needed to shed light on such an important issue. Lastly, we acknowledge that the wide age group that we chose could represent an issue. However, it should be noted that this was initially meant as a pilot study on infants and, considering the low number of infants undergoing blood sampling in our unit, we preferred to widen the age range to obtain a real-life study on a pediatric blood-drawing center with daily routine procedures carried out on children of all ages. For the same reasons, we did not provide formal sample size calculation. Nevertheless, we believe that evaluating individually the 3 age groups with specific tools reduced heterogeneity, giving strength to our work when compared with previous similar studies.

In conclusion, our study shows that the Buzzy device effectively reduces pain caused by percutaneous antecubital venipuncture in children of all ages and represents a useful, cheap, and easy-to-use strategy combining vibration and cold, which could be used in blood-drawing centers in routine needle-related procedures in busy medical settings.

## Supplementary Material
