# Supplementary figures and images for: The Effectiveness of the Buzzy Device in Reducing Pain in Children Undergoing Venipuncture: A Single-Center Experience
Source: Pediatr Emerg Care. 2023 Jul 22;39(10):760–5. doi: 10.1097/PEC.0000000000003011 (PMC10547103; doi:10.1097/PEC.0000000000003011)

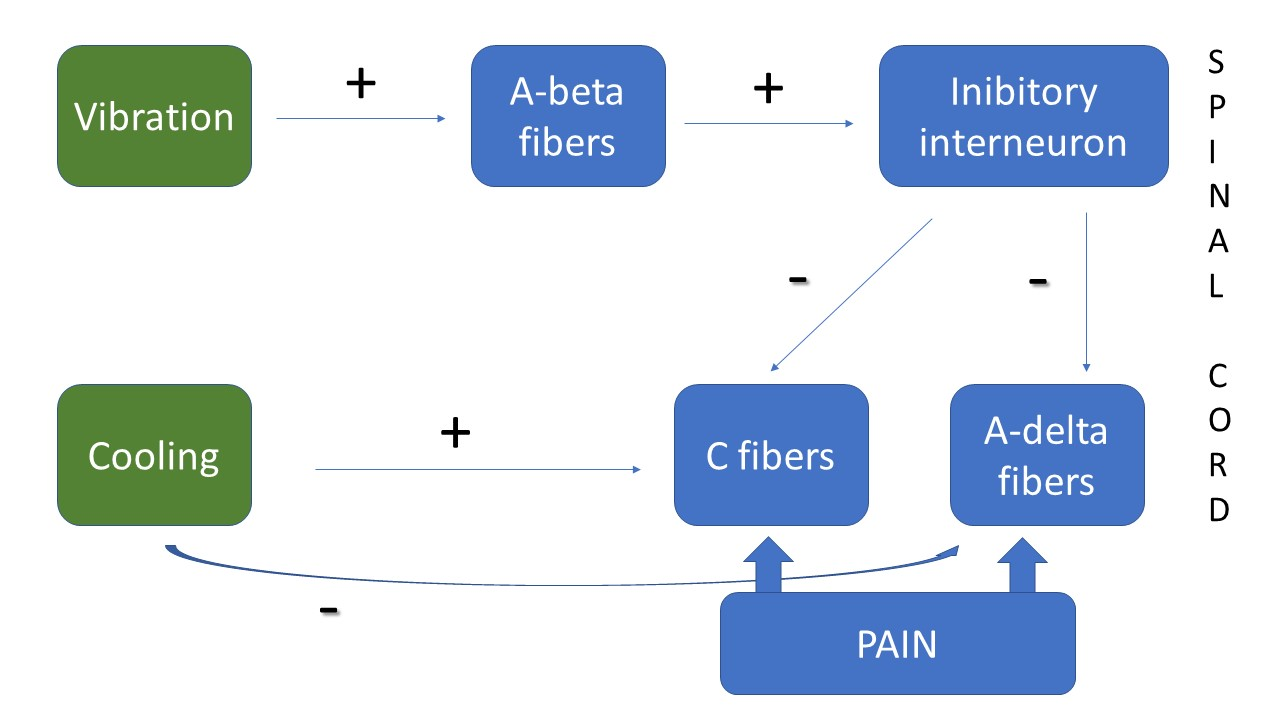

Supplement: Supplementary file 1 [file pcare-39-760-s001.tif]
